# A Dynamic Data-Driven Framework for Biological Data Using 2D Barcodes

**DOI:** 10.1155/2012/892098

**Published:** 2012-12-09

**Authors:** Hui Li, Chunmei Liu

**Affiliations:** Department of Systems and Computer Science, Howard University, Washington, DC 20059, USA

## Abstract

Biology data is increasing exponentially from biological laboratories. It is a complicated problem for further processing the data. Processing computational data and data from biological laboratories manually may lead to potential errors in further analysis. In this paper, we proposed an efficient data-driven framework to inspect laboratory equipment and reduce impending failures. Our method takes advantage of the 2D barcode technology which can be installed on the specimen as a trigger for the data-driven system. For this end, we proposed a series of algorithms to speed up the data processing. The results show that the proposed system increases the system's scalability and flexibility. Also, it demonstrates the ability of linking a physical object with digital information to reduce the manual work related to experimental specimen. The characteristics such as high capacity of storage and data management of the 2D barcode technology provide a solution to collect experimental laboratory data in a quick and accurate fashion.

## 1. Introduction

With the development of various biological techniques, the study of complex biological mechanism on the molecular level is close to disclose the reality of life. Biological experiment always provides the most powerful first-hand evidence of the changes or discovery of biological systems. Currently, conforming experimental cohort, sampling, tagging, freezing specimens, selecting experimental instrument, and generating experimental data are time consuming, error prone, and laboratory extensive for biologists. Particularly, the changes of the previous experimental schema will cause lots of unexpected errors or troubles. Data grows exponentially every day; a system solution is far from the utility. It is necessary and urgent to propose an efficient computational approach to systematically manage and simplify the whole process to improve biology data management and to eliminate potential errors as well as save time [1–3].

Efficiently managing experimental data is always the first demand for biologists. For instance, in most cases, biologists plan the experimental schema and detailed steps by hand before they start to do the experiments. Knowledge about the results to be obtained experimentally is also queried manually from online databases or websites. Once they finish the experiments, they have to tag manually the data generated from the experiments for further analyzing the data. At the same time, paper work to describe the details of the specimen and experimental schema has to be prepared and input manually into a computer, which may lead to some errors. In particular, the different biological specimen sampling methods and standards used in various experimental laboratories result in the difficulty of reproducibility of the experiments as well as exchanging data among different groups. With the availability of the computational recognition and image processing technology, the 2-dimensional (2D) barcode technologies provide an easy solution for the experiment management and data analysis, track, and management. 2D barcode is a type of automatic recognition method by attaching encoded information on the specimen and dataset [4]. 2D barcode is one of the popular techniques and widely applied in multiple fields such as products anti-counterfeit, map guidance, health system, and websites [5–7]. Although the method has been applied successfully in these fields, exchanging data among different groups is impossible for sharing data among different laboratories. Obviously, the inconsistence of the protocols between different phrases of the experiments increases the difficulty of the 2D barcode recognition system. Therefore, the implementation of this technology firstly needs the consistence and standard of protocols for data collection, biological specimen tagging, slides, and specimen containers.

Motivated by these challenges, we apply the 2D barcode technique and extend its ability to deal with experimental-related information to draft experimental schema, tag data, and store experimental protocol. We thus propose a data-driven laboratory information system which inspects laboratory equipment and eliminate impending failures. We put 2D barcodes on each specimen and input all the information in it which is to be recognized easily by any electronic scanner such as a mobile phone or a computer or any equipment with a camera. Also some image information can be stored in the 2D barcodes. The experiments show the proposed data-driven laboratory information management system based on the 2D barcode technique which improves the efficiency and reliability of the laboratory management system without the involvement of biologists.

The paper is organized as follows. The overview section introduces the proposed method where the 2D barcode technique is the key of this section. In the method section, we introduce a data-driven framework which can reduce the computational process and minimize total delay time. The results section shows the capability of the proposed method. In conclusion and algorithms section, we summarize the method.

## 2. Dynamic Data-Driven Framework for a Biological System

The concept of dynamic data-driven framework dynamically creates computational pipelines and is trigged by the biological data. Since the large volume of experiment data produced from the laboratories need to be processed for biologists, most of biology experimental dataset-related information is complex and heterogeneous. In order to integrate heterogeneous data, we use XML as the 2D encode barcode format. All the information is structured by using the XML format and we build an xml parsing model to parse and decode the 2D barcode images. 2D barcodes can be read by optical scanners with special software. In order to analyze the raw data, there are a computational pipeline and a database to analyze the raw data and store them, respectively. For our data-driven framework, each pipeline is created and trigged when it receives 2D barcode information.

### 2.1. Framework Model

**Figure 1:**
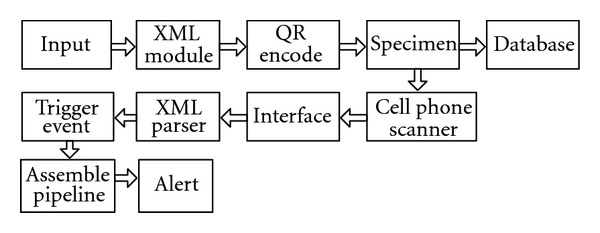
The model of the service-oriented dynamic data-driven framework.

The framework used in this paper is shown in Figure 1. The XML module transforms the input file into a QR support encoding format, and then the specimen is tagged by the 2D barcodes with the QR codes. The information of the 2D barcodes is saved into DBXML [8] at the same time. The event trigger is also written in the 2D barcodes. When the users scan the 2D barcodes and read the trigger event, the XML parser will parse the xml file to start the pipeline.

### 2.2. Application of the System to Biological Systems

In this section, we describe the data-driven framework on biological systems. The framework includes four main components. Figure 1 shows the four components used in the framework.

1. *The first part is the identification* of specimen using the 2D barcode technique. It collects all the detailed information of the specimen by a scanning device. The user will then get a list of all the barcodes and it also facilitates the interactions with the 2D barcode reader such as a smart phone.
2. *The second part is the pipeline module*; the data scanned by the 2D barcode is a trigger for driving the pipeline which directly obtains the required data and information.
3. *The third part is the detection module*, which will monitor and detect the congestion node of the pipeline and then alert the user the next data congestion point. If it finds any data need further experimental steps, it will then notify the experiment staff to finish the experiment.
4. *Using a heuristic algorithm t*o find paths of the pipeline by calculating the weights of nodes in the pipeline and trigger the most efficient pipeline.

## 3. Algorithms

In this paper, there are two types of dynamic biology data (raw and refer data) as mentioned in the above section. We first embed the information of a specimen item in a barcode and the information like the expiration date can be easily seen. In order to avoid the congestion of the pipeline and find the path out of the pipeline, we proposed a heuristic algorithm and assembling pipeline strategy.

When a biologist parses the XML and checks the data completeness, he first checks the entire list of the specimen scanned and finds the node of the pipeline can be triggered and dynamically assembles the pipeline. The heuristic algorithm ensures the above steps to be included sequentially. The detailed description of the algorithm is as follows.

**Step 1 (constructing the data-driven mechanism)**

**Figure 2:**
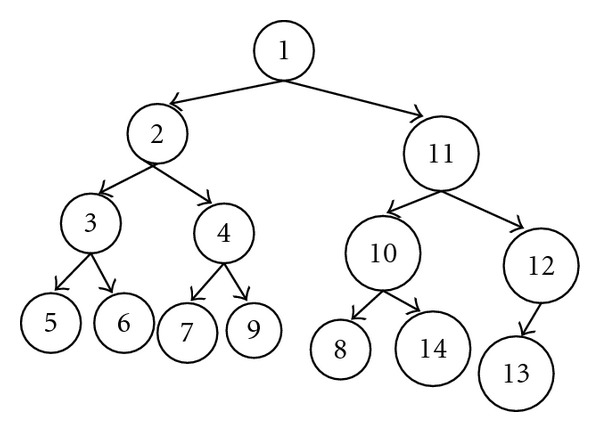
The process of the path search.

Providing the standard formatted specimen information to generate a 2D barcode image as shown in Figure 2, 2D barcodes save the information of the specimen to be identified. In this paper, we use the XML format to encode the 2D barcodes for experimental specimen information. We define the nodes of the pipeline and the triggering event which are embedded in the 2D barcodes. The nodes of the pipeline include *R* functions, raw data uploading, data preprocessing, and Matlab functions. The pipeline starts from the event processing. The operations include data verification, paths of pipeline checking and pipeline assembly. Since 2D barcodes contain considerably less than 2000 characters, we divide the 2D barcode data into two categories: (1)  *raw data* which means all the data from biological experiments can be stored in the barcode and (2)  *refer data* which indicates that the data of the experiment exceeds the limit of the 2D barcode, and then the 2D barcode represents the description of the raw data and the location of the data for external database. In this paper, we use a XML file for encoding information inside a 2D barcode. We will describe the format that is generated for specific purposes shown in experiment data format (Algorithm  1).

**Step 2 (pipeline construction)**

The second step is to define the node of the pipeline in the system and determine the weight value for each node according to the data and the final goal. The pipeline is built based on the data and bioinformatics program analysis and the pipeline consists of in-house programs, raw data from the experiment, intermediate results, and tools, which are illustrated in description of algorithm (Algorithm  2).

We assume that the pipeline congestion is caused by lacking of data or waiting for other processing and each node relies on the output of the previous node in the pipeline tree. Each node has four possible directions (left, right, up, and down). Consequently, the raw data of the node can be moved along to a goal node. The weighted value of the node is defined according to the following:

(1) E time(i,j)=∑1mDt(k)+Dis(i),

where *i* and *j* are the sequence number representations of the node in the pipeline tree, *D*_*t*_(*k*) is the waiting time for the data that the node requires, and *m* is the number of required data between the node *i* and node *j*.

**Step 3 (assemble a pipeline)**

The aim of this step is to obtain the effective pipeline tree for the raw data. In order to minimize total delay time for the computational process of the pipeline, we use the Dijkstra algorithm [9] to calculate the accumulative weights and find an efficient pipeline out of all possible nodes. In the Dijkstra process, we select the highest priority node and move it from the raw data node to the goal node and obtain a shortest path by calculating the value in the pipeline tree. The algorithm will output a pipeline tree and alert users about the lack of data.

## 4. Experiments

In order to test the performance of our framework, we generate simulation data for the pipeline, and then used our method to run Table 1 shows the simulation results from the raw data. In each experiment, Dijkstra approaches have been executed for searching the most efficient pipeline tree based on the trigger event. If the results of two prediction strategies have the same accuracy and efficiency, the difference between these average travelling times should be close to zero.

## 5. Conclusion

In this paper, we propose a data-driven framework to improve the management efficiency of the biology laboratory system. It encodes the detailed information of a specimen in the 2D barcodes and embeds the trigger event for a pipeline. The experimental results show that the proposed method improves system efficiency by processing a fraction of a large volume of data. It not only promotes the efficiency of the biological system, but also reduces the error of the system.

## Figures and Tables

**Algorithm 1:**
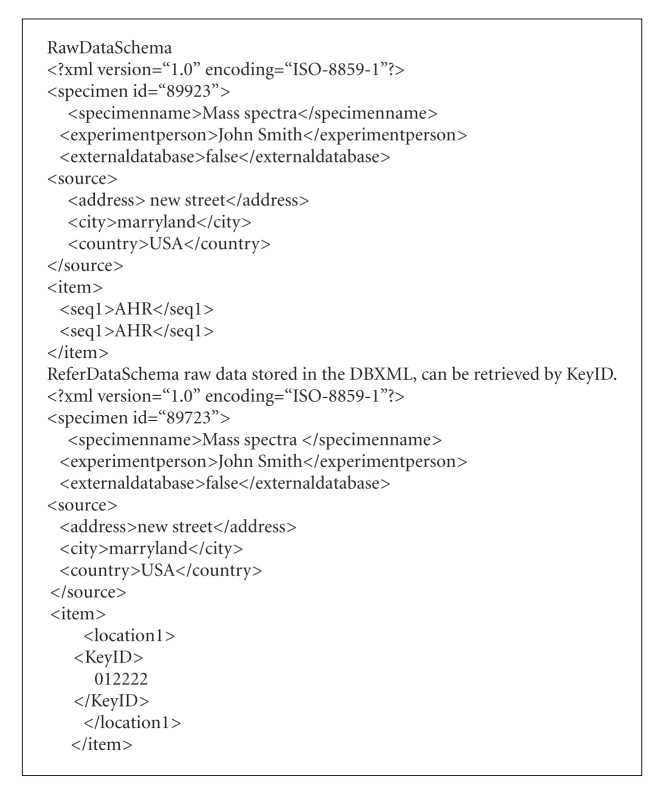
Experiment data format.

**Algorithm 2:**
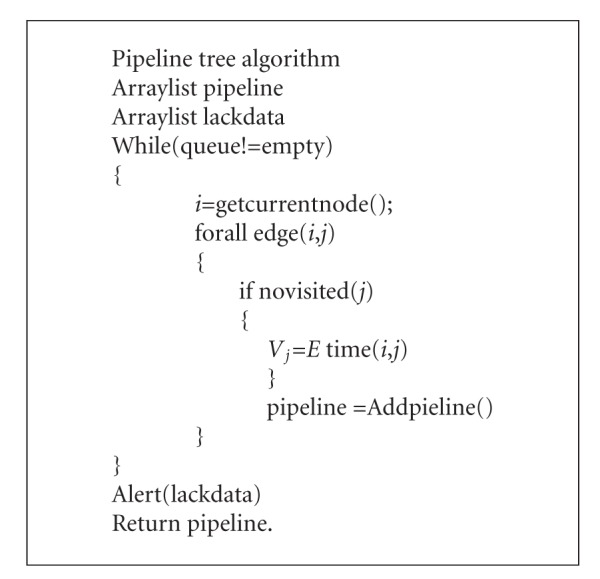
Description of algorithm.

**Table 1:** The simulation results of the data-driven framework.

Number of nodes	Data-driven	No data-driven
of a pipeline	framework	framework
40	Dijkstra	No algorithm
	15 hours	21 hours
	Improvement (21−15)	
	hours/21 = 28.57%	
